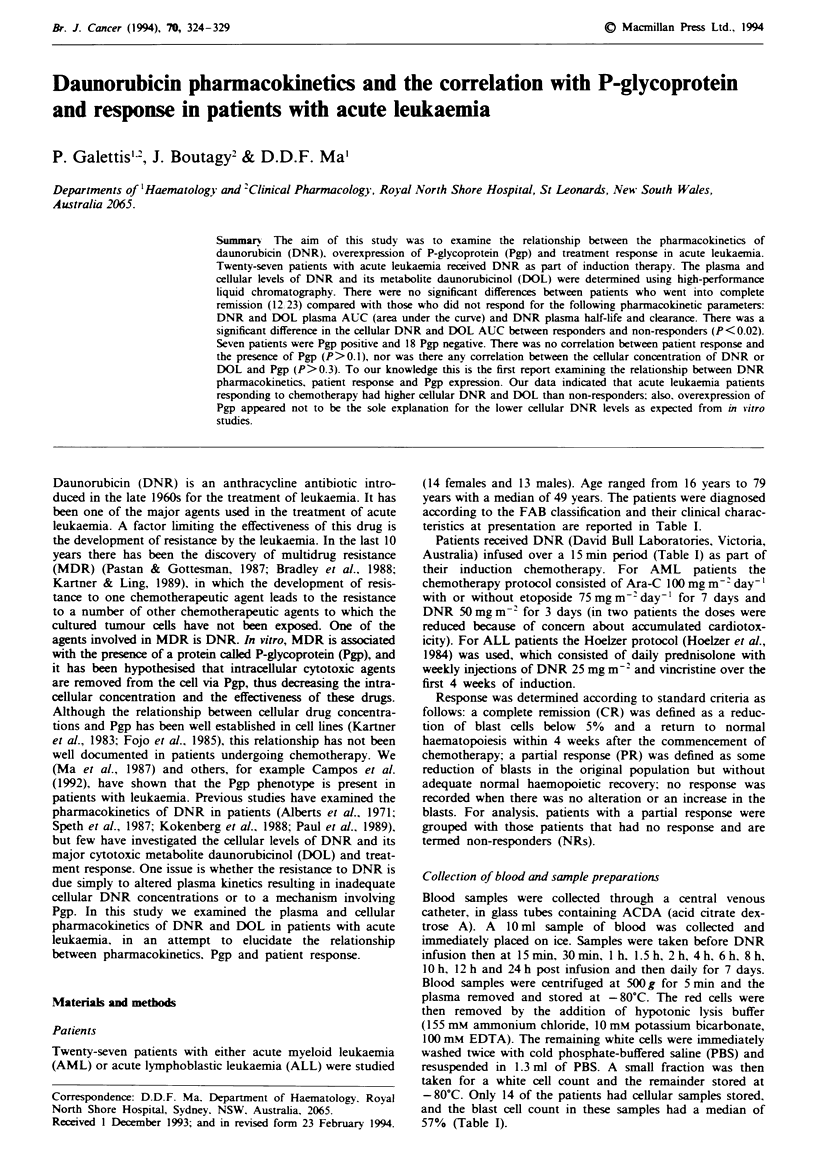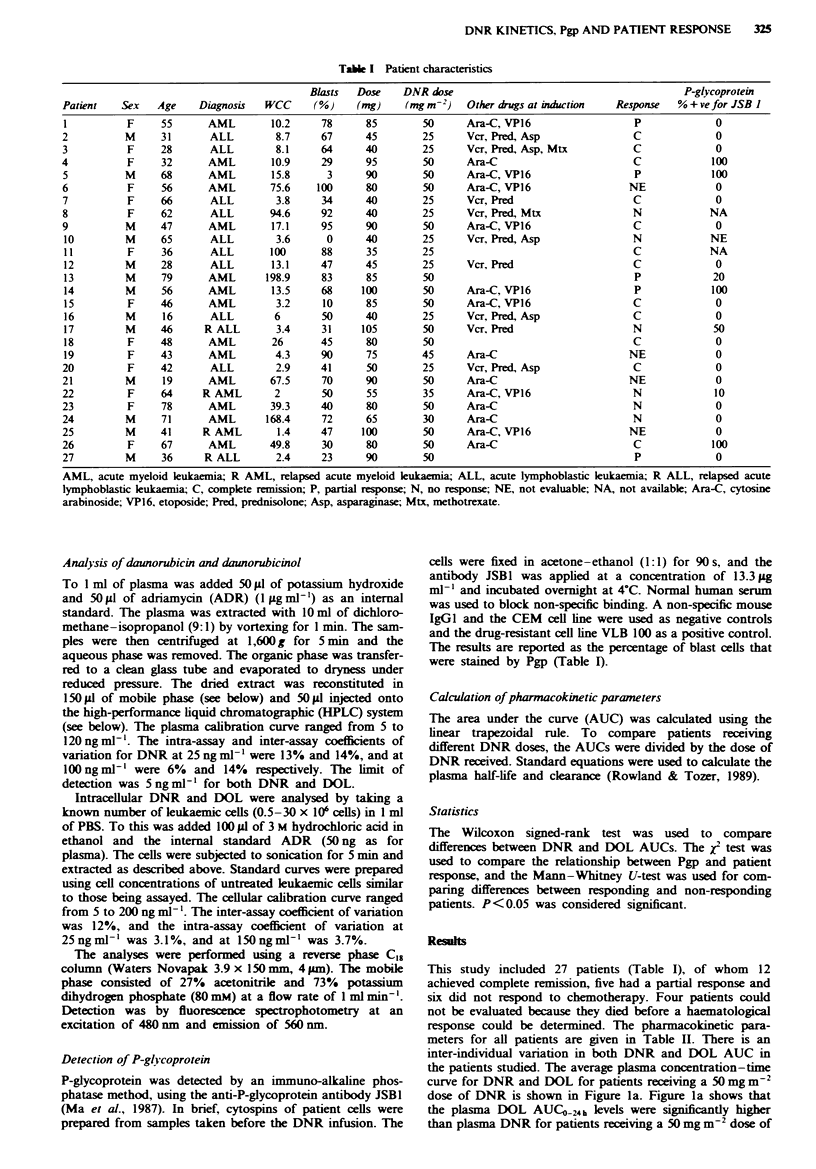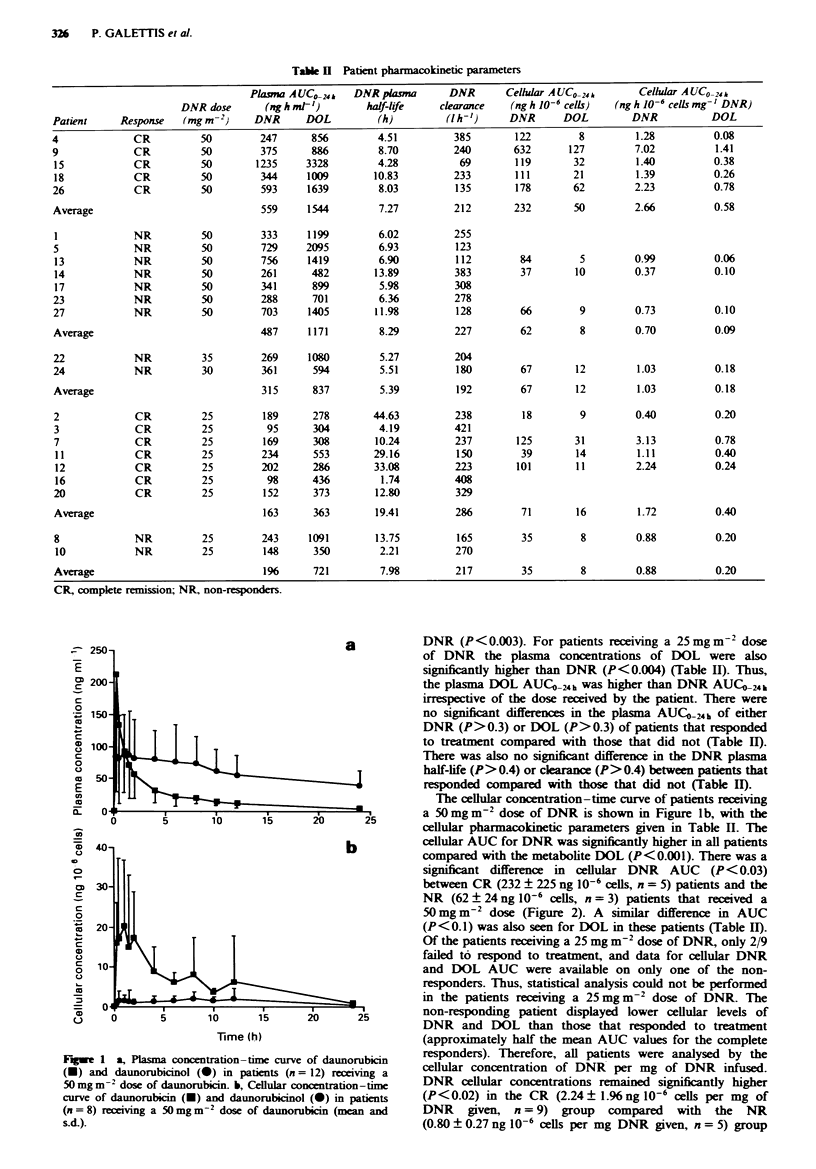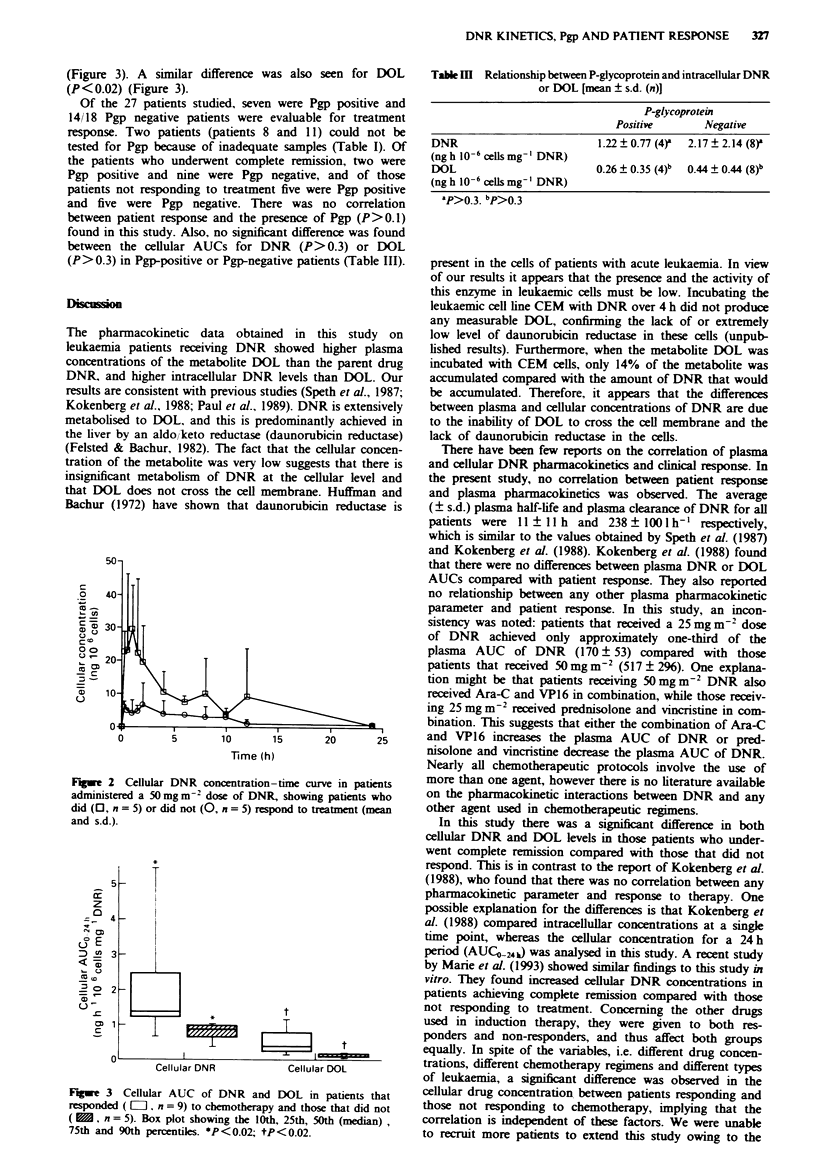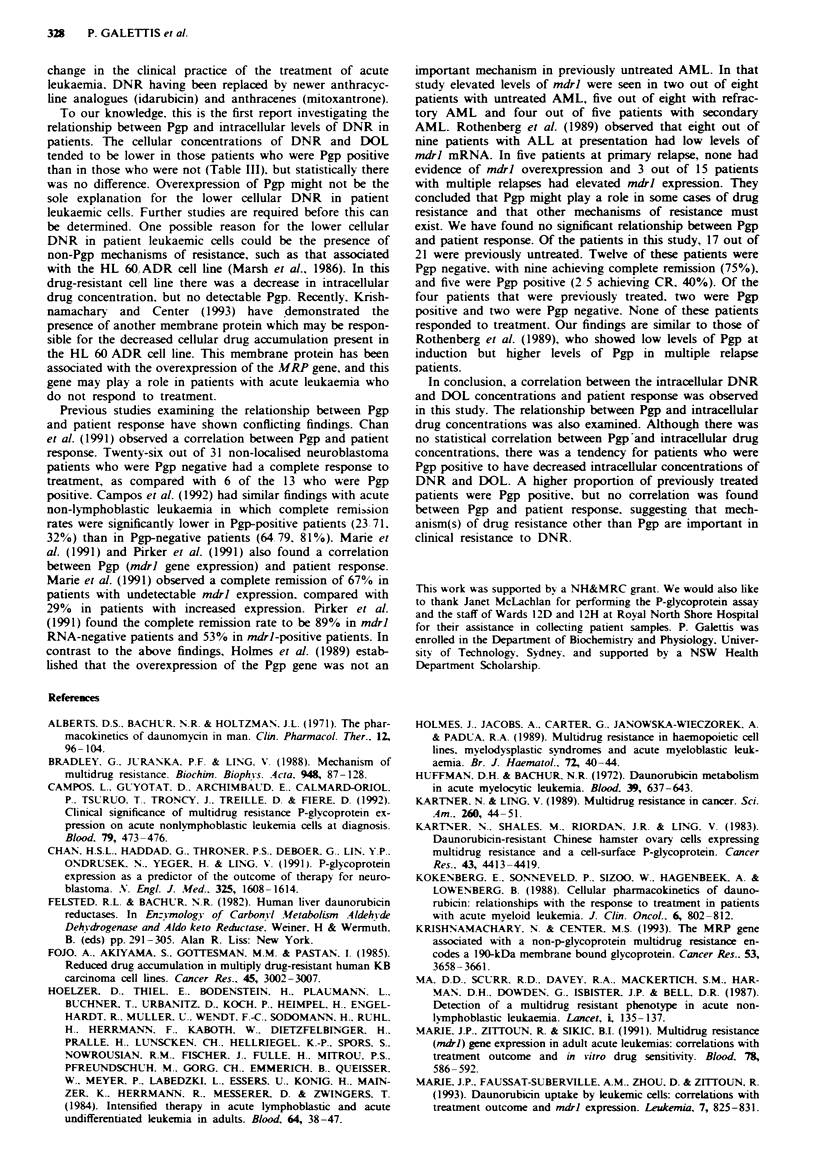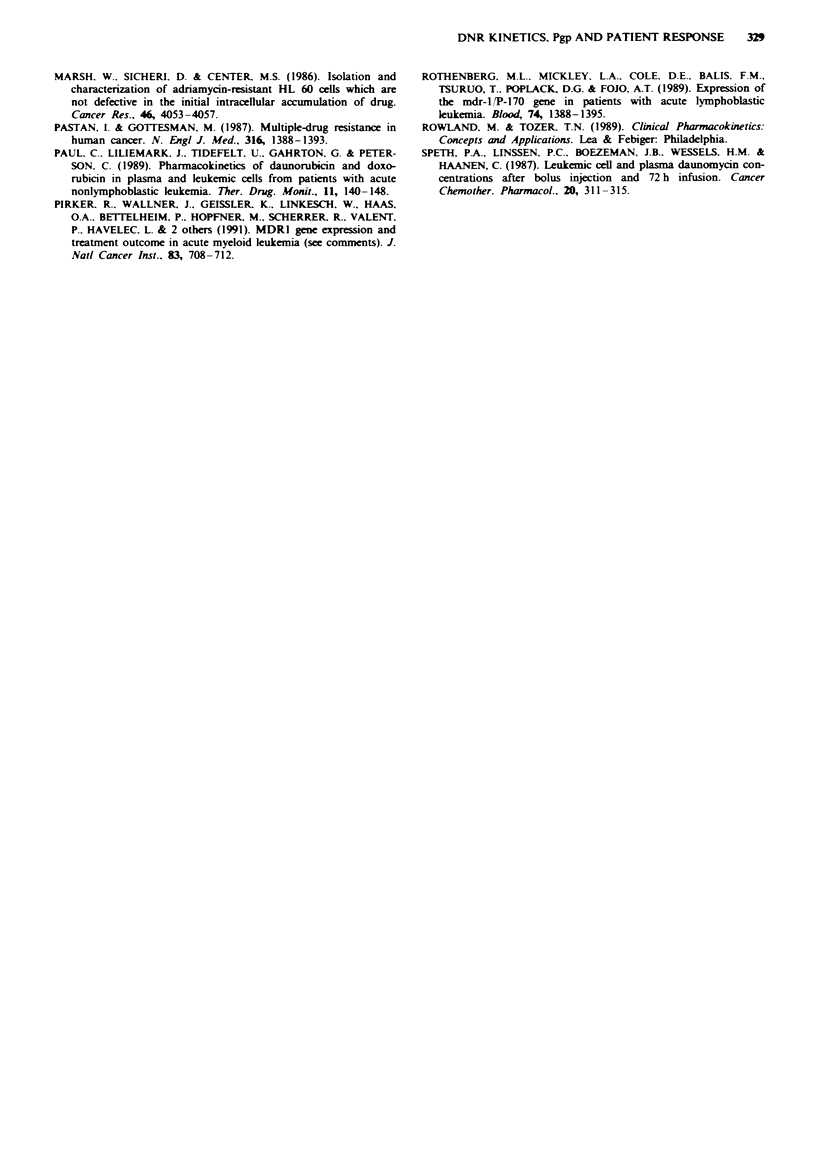# Daunorubicin pharmacokinetics and the correlation with P-glycoprotein and response in patients with acute leukaemia.

**DOI:** 10.1038/bjc.1994.301

**Published:** 1994-08

**Authors:** P. Galettis, J. Boutagy, D. D. Ma

**Affiliations:** Department of Haematology, Royal North Shore Hospital, St Leonards, New South Wales, Australia.

## Abstract

The aim of this study was to examine the relationship between the pharmacokinetics of daunorubicin (DNR), overexpression of P-glycoprotein (Pgp) and treatment response in acute leukaemia. Twenty-seven patients with acute leukaemia received DNR as part of induction therapy. The plasma and cellular levels of DNR and its metabolite daunorubicinol (DOL) were determined using high-performance liquid chromatography. There were no significant differences between patients who went into complete remission (12/23) compared with those who did not respond for the following pharmacokinetic parameters: DNR and DOL plasma AUC (area under the curve) and DNR plasma half-life and clearance. There was a significant difference in the cellular DNR and DOL AUC between responders and non-responders (P < 0.02). Seven patients were Pgp positive and 18 Pgp negative. There was no correlation between patient response and the presence of Pgp (P > 0.1), nor was there any correlation between the cellular concentration of DNR or DOL and Pgp (P > 0.3). To our knowledge this is the first report examining the relationship between DNR pharmacokinetics, patient response and Pgp expression. Our data indicated that acute leukaemia patients responding to chemotherapy had higher cellular DNR and DOL than non-responders; also, overexpression of Pgp appeared not to be the sole explanation for the lower cellular DNR levels as expected from in vitro studies.


Br. J. Cancer (1994), 70, 324-329 © Macmillan Press Ltd., 1994

### Daunorubicin pharmacokinetics and the correlation with P-glycoprotein and response in patients with acute leukaemia

P. Galettis[1,2], J. Boutagy[2] & D.D.F. Ma[1]

*Departments of [1]Haematology and [2]Clinical Pharmacology, Royal North Shore Hospital, St Leonards, New South Wales, Australia 2065.*

**Summary** The aim of this study was to examine the relationship between the pharmacokinetics of daunorubicin (DNR), overexpression of P-glycoprotein (Pgp) and treatment response in acute leukaemia. Twenty-seven patients with acute leukaemia received DNR as part of induction therapy. The plasma and cellular levels of DNR and its metabolite daunorubicinol (DOL) were determined using high-performance liquid chromatography. There were no significant differences between patients who went into complete remission (12/23) compared with those who did not respond for the following pharmacokinetic parameters: DNR and DOL plasma AUC (area under the curve) and DNR plasma half-life and clearance. There was a significant difference in the cellular DNR and DOL AUC between responders and non-responders ($P<0.02$). Seven patients were Pgp positive and 18 Pgp negative. There was no correlation between patient response and the presence of Pgp ($P>0.1$), nor was there any correlation between the cellular concentration of DNR or DOL and Pgp ($P>0.3$). To our knowledge this is the first report examining the relationship between DNR pharmacokinetics, patient response and Pgp expression. Our data indicated that acute leukaemia patients responding to chemotherapy had higher cellular DNR and DOL than non-responders; also, overexpression of Pgp appeared not to be the sole explanation for the lower cellular DNR levels as expected from *in vitro* studies.

Daunorubicin (DNR) is an anthracycline antibiotic introduced in the late 1960s for the treatment of leukaemia. It has been one of the major agents used in the treatment of acute leukaemia. A factor limiting the effectiveness of this drug is the development of resistance by the leukaemia. In the last 10 years there has been the discovery of multidrug resistance (MDR) (Pastan & Gottesman, 1987; Bradley *et al.*, 1988; Kartner & Ling, 1989), in which the development of resistance to one chemotherapeutic agent leads to the resistance to a number of other chemotherapeutic agents to which the cultured tumour cells have not been exposed. One of the agents involved in MDR is DNR. *In vitro*, MDR is associated with the presence of a protein called P-glycoprotein (Pgp), and it has been hypothesised that intracellular cytotoxic agents are removed from the cell via Pgp, thus decreasing the intracellular concentration and the effectiveness of these drugs. Although the relationship between cellular drug concentrations and Pgp has been well established in cell lines (Kartner *et al.*, 1983; Fojo *et al.*, 1985), this relationship has not been well documented in patients undergoing chemotherapy. We (Ma *et al.*, 1987) and others, for example Campos *et al.* (1992), have shown that the Pgp phenotype is present in patients with leukaemia. Previous studies have examined the pharmacokinetics of DNR in patients (Alberts *et al.*, 1971; Speth *et al.*, 1987; Kokenberg *et al.*, 1988; Paul *et al.*, 1989), but few have investigated the cellular levels of DNR and its major cytotoxic metabolite daunorubicinol (DOL) and treatment response. One issue is whether the resistance to DNR is due simply to altered plasma kinetics resulting in inadequate cellular DNR concentrations or to a mechanism involving Pgp. In this study we examined the plasma and cellular pharmacokinetics of DNR and DOL in patients with acute leukaemia, in an attempt to elucidate the relationship between pharmacokinetics, Pgp and patient response.

### Materials and methods

### *Patients*

Twenty-seven patients with either acute myeloid leukaemia (AML) or acute lymphoblastic leukaemia (ALL) were studied (14 females and 13 males). Age ranged from 16 years to 79 years with a median of 49 years. The patients were diagnosed according to the FAB classification and their clinical characteristics at presentation are reported in Table I.

Patients received DNR (David Bull Laboratories, Victoria, Australia) infused over a 15 min period (Table I) as part of their induction chemotherapy. For AML patients the chemotherapy protocol consisted of Ara-C 100 mg $m^{-2}$ $day^{-1}$ with or without etoposide 75 mg $m^{-2}$ $day^{-1}$ for 7 days and DNR 50 mg $m^{-2}$ for 3 days (in two patients the doses were reduced because of concern about accumulated cardiotoxicity). For ALL patients the Hoelzer protocol (Hoelzer *et al.*, 1984) was used, which consisted of daily prednisolone with weekly injections of DNR 25 mg $m^{-2}$ and vincristine over the first 4 weeks of induction.

Response was determined according to standard criteria as follows: a complete remission (CR) was defined as a reduction of blast cells below 5% and a return to normal haematopoiesis within 4 weeks after the commencement of chemotherapy; a partial response (PR) was defined as some reduction of blasts in the original population but without adequate normal haemopoietic recovery; no response was recorded when there was no alteration or an increase in the blasts. For analysis, patients with a partial response were grouped with those patients that had no response and are termed non-responders (NRs).

### *Collection of blood and sample preparations*

Blood samples were collected through a central venous catheter, in glass tubes containing ACDA (acid citrate dextrose A). A 10 ml sample of blood was collected and immediately placed on ice. Samples were taken before DNR infusion then at 15 min, 30 min, 1 h, 1.5 h, 2 h, 4 h, 6 h, 8 h, 10 h, 12 h and 24 h post infusion and then daily for 7 days. Blood samples were centrifuged at 500 *g* for 5 min and the plasma removed and stored at $-80°C$. The red cells were then removed by the addition of hypotonic lysis buffer (155 mM ammonium chloride, 10 mM potassium bicarbonate, 100 mM EDTA). The remaining white cells were immediately washed twice with cold phosphate-buffered saline (PBS) and resuspended in 1.3 ml of PBS. A small fraction was then taken for a white cell count and the remainder stored at $-80°C$. Only 14 of the patients had cellular samples stored, and the blast cell count in these samples had a median of 57% (Table I).

Correspondence: D.D.F. Ma, Department of Haematology, Royal North Shore Hospital, Sydney, NSW, Australia, 2065.
Received 1 December 1993; and in revised form 23 February 1994.

**Table I** Patient characteristics

| Patient | Sex | Age | Diagnosis | WCC | Blasts (%) | Dose (mg) | DNR dose (mg m$^{-2}$) | Other drugs at induction | Response | P-glycoprotein % + ve for JSB 1 |
|---|---|---|---|---|---|---|---|---|---|---|
| 1 | F | 55 | AML | 10.2 | 78 | 85 | 50 | Ara-C, VP16 | P | 0 |
| 2 | M | 31 | ALL | 8.7 | 67 | 45 | 25 | Vcr, Pred, Asp | C | 0 |
| 3 | F | 28 | ALL | 8.1 | 64 | 40 | 25 | Vcr, Pred, Asp, Mtx | C | 0 |
| 4 | F | 32 | AML | 10.9 | 29 | 95 | 50 | Ara-C | C | 100 |
| 5 | M | 68 | AML | 15.8 | 3 | 90 | 50 | Ara-C, VP16 | P | 100 |
| 6 | F | 56 | AML | 75.6 | 100 | 80 | 50 | Ara-C, VP16 | NE | 0 |
| 7 | F | 66 | ALL | 3.8 | 34 | 40 | 25 | Vcr, Pred | C | 0 |
| 8 | F | 62 | ALL | 94.6 | 92 | 40 | 25 | Vcr, Pred, Mtx | N | NA |
| 9 | M | 47 | AML | 17.1 | 95 | 90 | 50 | Ara-C, VP16 | C | 0 |
| 10 | M | 65 | ALL | 3.6 | 0 | 40 | 25 | Vcr, Pred, Asp | N | NE |
| 11 | F | 36 | ALL | 100 | 88 | 35 | 25 | | C | NA |
| 12 | M | 28 | ALL | 13.1 | 47 | 45 | 25 | Vcr, Pred | C | 0 |
| 13 | M | 79 | AML | 198.9 | 83 | 85 | 50 | | P | 20 |
| 14 | M | 56 | AML | 13.5 | 68 | 100 | 50 | Ara-C, VP16 | P | 100 |
| 15 | F | 46 | AML | 3.2 | 10 | 85 | 50 | Ara-C, VP16 | C | 0 |
| 16 | M | 16 | ALL | 6 | 50 | 40 | 25 | Vcr, Pred, Asp | C | 0 |
| 17 | M | 46 | R ALL | 3.4 | 31 | 105 | 50 | Vcr, Pred | N | 50 |
| 18 | F | 48 | AML | 26 | 45 | 80 | 50 | | C | 0 |
| 19 | F | 43 | AML | 4.3 | 90 | 75 | 45 | Ara-C | NE | 0 |
| 20 | F | 42 | ALL | 2.9 | 41 | 50 | 25 | Vcr, Pred, Asp | C | 0 |
| 21 | M | 19 | AML | 67.5 | 70 | 90 | 50 | Ara-C | NE | 0 |
| 22 | F | 64 | R AML | 2 | 50 | 55 | 35 | Ara-C, VP16 | N | 10 |
| 23 | F | 78 | AML | 39.3 | 40 | 80 | 50 | Ara-C | N | 0 |
| 24 | M | 71 | AML | 168.4 | 72 | 65 | 30 | Ara-C | N | 0 |
| 25 | M | 41 | R AML | 1.4 | 47 | 100 | 50 | Ara-C, VP16 | NE | 0 |
| 26 | F | 67 | AML | 49.8 | 30 | 80 | 50 | Ara-C | C | 100 |
| 27 | M | 36 | R ALL | 2.4 | 23 | 90 | 50 | | P | 0 |

AML, acute myeloid leukaemia; R AML, relapsed acute myeloid leukaemia; ALL, acute lymphoblastic leukaemia; R ALL, relapsed acute lymphoblastic leukaemia; C, complete remission; P, partial response; N, no response; NE, not evaluable; NA, not available; Ara-C, cytosine arabinoside; VP16, etoposide; Pred, prednisolone; Asp, asparaginase; Mtx, methotrexate.

### *Analysis of daunorubicin and daunorubicinol*

To 1 ml of plasma was added 50 µl of potassium hydroxide and 50 µl of adriamycin (ADR) (1 µg ml$^{-1}$) as an internal standard. The plasma was extracted with 10 ml of dichloromethane-isopropanol (9:1) by vortexing for 1 min. The samples were then centrifuged at 1,600 *g* for 5 min and the aqueous phase was removed. The organic phase was transferred to a clean glass tube and evaporated to dryness under reduced pressure. The dried extract was reconstituted in 150 µl of mobile phase (see below) and 50 µl injected onto the high-performance liquid chromatographic (HPLC) system (see below). The plasma calibration curve ranged from 5 to 120 ng ml$^{-1}$. The intra-assay and inter-assay coefficients of variation for DNR at 25 ng ml$^{-1}$ were 13% and 14%, and at 100 ng ml$^{-1}$ were 6% and 14% respectively. The limit of detection was 5 ng ml$^{-1}$ for both DNR and DOL.

Intracellular DNR and DOL were analysed by taking a known number of leukaemic cells (0.5–30 × $10^6$ cells) in 1 ml of PBS. To this was added 100 µl of 3 M hydrochloric acid in ethanol and the internal standard ADR (50 ng as for plasma). The cells were subjected to sonication for 5 min and extracted as described above. Standard curves were prepared using cell concentrations of untreated leukaemic cells similar to those being assayed. The cellular calibration curve ranged from 5 to 200 ng ml$^{-1}$. The inter-assay coefficient of variation was 12%, and the intra-assay coefficient of variation at 25 ng ml$^{-1}$ was 3.1%, and at 150 ng ml$^{-1}$ was 3.7%.

The analyses were performed using a reverse phase $C_{18}$ column (Waters Novapak 3.9 × 150 mm, 4 µm). The mobile phase consisted of 27% acetonitrile and 73% potassium dihydrogen phosphate (80 mM) at a flow rate of 1 ml min$^{-1}$. Detection was by fluorescence spectrophotometry at an excitation of 480 nm and emission of 560 nm.

### *Detection of P-glycoprotein*

P-glycoprotein was detected by an immuno-alkaline phosphatase method, using the anti-P-glycoprotein antibody JSB1 (Ma *et al.*, 1987). In brief, cytospins of patient cells were prepared from samples taken before the DNR infusion. The cells were fixed in acetone-ethanol (1:1) for 90 s, and the antibody JSB1 was applied at a concentration of 13.3 µg ml$^{-1}$ and incubated overnight at 4°C. Normal human serum was used to block non-specific binding. A non-specific mouse IgG1 and the CEM cell line were used as negative controls and the drug-resistant cell line VLB 100 as a positive control. The results are reported as the percentage of blast cells that were stained by Pgp (Table I).

### *Calculation of pharmacokinetic parameters*

The area under the curve (AUC) was calculated using the linear trapezoidal rule. To compare patients receiving different DNR doses, the AUCs were divided by the dose of DNR received. Standard equations were used to calculate the plasma half-life and clearance (Rowland & Tozer, 1989).

### *Statistics*

The Wilcoxon signed-rank test was used to compare differences between DNR and DOL AUCs. The $\chi^2$ test was used to compare the relationship between Pgp and patient response, and the Mann-Whitney *U*-test was used for comparing differences between responding and non-responding patients. $P < 0.05$ was considered significant.

### Results

This study included 27 patients (Table I), of whom 12 achieved complete remission, five had a partial response and six did not respond to chemotherapy. Four patients could not be evaluated because they died before a haematological response could be determined. The pharmacokinetic parameters for all patients are given in Table II. There is an inter-individual variation in both DNR and DOL AUC in the patients studied. The average plasma concentration-time curve for DNR and DOL for patients receiving a 50 mg m$^{-2}$ dose of DNR is shown in Figure 1a. Figure 1a shows that the plasma DOL $AUC_{0-24h}$ levels were significantly higher than plasma DNR for patients receiving a 50 mg m$^{-2}$ dose of

**Table II** Patient pharmacokinetic parameters

| Patient | Response | DNR dose ($mg\ m^{-2}$) | Plasma $AUC_{0-24h}$ ($ng\ h\ ml^{-1}$) DNR | DOL | DNR plasma half-life (h) | DNR clearance ($l\ h^{-1}$) | Cellular $AUC_{0-24h}$ ($ng\ h\ 10^{-6}$ cells) DNR | DOL | Cellular $AUC_{0-24h}$ ($ng\ h\ 10^{-6}$ cells $mg^{-1}$ DNR) DNR | DOL |
|---|---|---|---|---|---|---|---|---|---|---|
| 4 | CR | 50 | 247 | 856 | 4.51 | 385 | 122 | 8 | 1.28 | 0.08 |
| 9 | CR | 50 | 375 | 886 | 8.70 | 240 | 632 | 127 | 7.02 | 1.41 |
| 15 | CR | 50 | 1235 | 3328 | 4.28 | 69 | 119 | 32 | 1.40 | 0.38 |
| 18 | CR | 50 | 344 | 1009 | 10.83 | 233 | 111 | 21 | 1.39 | 0.26 |
| 26 | CR | 50 | 593 | 1639 | 8.03 | 135 | 178 | 62 | 2.23 | 0.78 |
| Average | | | 559 | 1544 | 7.27 | 212 | 232 | 50 | 2.66 | 0.58 |
| 1 | NR | 50 | 333 | 1199 | 6.02 | 255 | | | | |
| 5 | NR | 50 | 729 | 2095 | 6.93 | 123 | | | | |
| 13 | NR | 50 | 756 | 1419 | 6.90 | 112 | 84 | 5 | 0.99 | 0.06 |
| 14 | NR | 50 | 261 | 482 | 13.89 | 383 | 37 | 10 | 0.37 | 0.10 |
| 17 | NR | 50 | 341 | 899 | 5.98 | 308 | | | | |
| 23 | NR | 50 | 288 | 701 | 6.36 | 278 | | | | |
| 27 | NR | 50 | 703 | 1405 | 11.98 | 128 | 66 | 9 | 0.73 | 0.10 |
| Average | | | 487 | 1171 | 8.29 | 227 | 62 | 8 | 0.70 | 0.09 |
| 22 | NR | 35 | 269 | 1080 | 5.27 | 204 | | | | |
| 24 | NR | 30 | 361 | 594 | 5.51 | 180 | 67 | 12 | 1.03 | 0.18 |
| Average | | | 315 | 837 | 5.39 | 192 | 67 | 12 | 1.03 | 0.18 |
| 2 | CR | 25 | 189 | 278 | 44.63 | 238 | 18 | 9 | 0.40 | 0.20 |
| 3 | CR | 25 | 95 | 304 | 4.19 | 421 | | | | |
| 7 | CR | 25 | 169 | 308 | 10.24 | 237 | 125 | 31 | 3.13 | 0.78 |
| 11 | CR | 25 | 234 | 553 | 29.16 | 150 | 39 | 14 | 1.11 | 0.40 |
| 12 | CR | 25 | 202 | 286 | 33.08 | 223 | 101 | 11 | 2.24 | 0.24 |
| 16 | CR | 25 | 98 | 436 | 1.74 | 408 | | | | |
| 20 | CR | 25 | 152 | 373 | 12.80 | 329 | | | | |
| Average | | | 163 | 363 | 19.41 | 286 | 71 | 16 | 1.72 | 0.40 |
| 8 | NR | 25 | 243 | 1091 | 13.75 | 165 | 35 | 8 | 0.88 | 0.20 |
| 10 | NR | 25 | 148 | 350 | 2.21 | 270 | | | | |
| Average | | | 196 | 721 | 7.98 | 217 | 35 | 8 | 0.88 | 0.20 |

CR, complete remission; NR, non-responders.

**Figure 1** **a**, Plasma concentration–time curve of daunorubicin (■) and daunorubicinol (●) in patients ($n = 12$) receiving a $50\ mg\ m^{-2}$ dose of daunorubicin. **b**, Cellular concentration–time curve of daunorubicin (■) and daunorubicinol (●) in patients ($n = 8$) receiving a $50\ mg\ m^{-2}$ dose of daunorubicin (mean and s.d.).

DNR ($P < 0.003$). For patients receiving a $25\ mg\ m^{-2}$ dose of DNR the plasma concentrations of DOL were also significantly higher than DNR ($P < 0.004$) (Table II). Thus, the plasma DOL $AUC_{0-24h}$ was higher than DNR $AUC_{0-24h}$ irrespective of the dose received by the patient. There were no significant differences in the plasma $AUC_{0-24h}$ of either DNR ($P > 0.3$) or DOL ($P > 0.3$) of patients that responded to treatment compared with those that did not (Table II). There was also no significant difference in the DNR plasma half-life ($P > 0.4$) or clearance ($P > 0.4$) between patients that responded compared with those that did not (Table II).

The cellular concentration–time curve of patients receiving a $50\ mg\ m^{-2}$ dose of DNR is shown in Figure 1b, with the cellular pharmacokinetic parameters given in Table II. The cellular AUC for DNR was significantly higher in all patients compared with the metabolite DOL ($P < 0.001$). There was a significant difference in cellular DNR AUC ($P < 0.03$) between CR ($232 \pm 225\ ng\ 10^{-6}$ cells, $n = 5$) patients and the NR ($62 \pm 24\ ng\ 10^{-6}$ cells, $n = 3$) patients that received a $50\ mg\ m^{-2}$ dose (Figure 2). A similar difference in AUC ($P < 0.1$) was also seen for DOL in these patients (Table II). Of the patients receiving a $25\ mg\ m^{-2}$ dose of DNR, only 2/9 failed to respond to treatment, and data for cellular DNR and DOL AUC were available on only one of the non-responders. Thus, statistical analysis could not be performed in the patients receiving a $25\ mg\ m^{-2}$ dose of DNR. The non-responding patient displayed lower cellular levels of DNR and DOL than those that responded to treatment (approximately half the mean AUC values for the complete responders). Therefore, all patients were analysed by the cellular concentration of DNR per mg of DNR infused. DNR cellular concentrations remained significantly higher ($P < 0.02$) in the CR ($2.24 \pm 1.96\ ng\ 10^{-6}$ cells per mg of DNR given, $n = 9$) group compared with the NR ($0.80 \pm 0.27\ ng\ 10^{-6}$ cells per mg DNR given, $n = 5$) group

(Figure 3). A similar difference was also seen for DOL ($P<0.02$) (Figure 3).

Of the 27 patients studied, seven were Pgp positive and 14/18 Pgp negative patients were evaluable for treatment response. Two patients (patients 8 and 11) could not be tested for Pgp because of inadequate samples (Table I). Of the patients who underwent complete remission, two were Pgp positive and nine were Pgp negative, and of those patients not responding to treatment five were Pgp positive and five were Pgp negative. There was no correlation between patient response and the presence of Pgp ($P>0.1$) found in this study. Also, no significant difference was found between the cellular AUCs for DNR ($P>0.3$) or DOL ($P>0.3$) in Pgp-positive or Pgp-negative patients (Table III).

### Discussion

The pharmacokinetic data obtained in this study on leukaemia patients receiving DNR showed higher plasma concentrations of the metabolite DOL than the parent drug DNR, and higher intracellular DNR levels than DOL. Our results are consistent with previous studies (Speth *et al.*, 1987; Kokenberg *et al.*, 1988; Paul *et al.*, 1989). DNR is extensively metabolised to DOL, and this is predominantly achieved in the liver by an aldo/keto reductase (daunorubicin reductase) (Felsted & Bachur, 1982). The fact that the cellular concentration of the metabolite was very low suggests that there is insignificant metabolism of DNR at the cellular level and that DOL does not cross the cell membrane. Huffman and Bachur (1972) have shown that daunorubicin reductase is

**Figure 2** Cellular DNR concentration-time curve in patients administered a 50 mg $m^{-2}$ dose of DNR, showing patients who did (□, $n = 5$) or did not (O, $n = 5$) respond to treatment (mean and s.d.).

**Figure 3** Cellular AUC of DNR and DOL in patients that responded (□, $n = 9$) to chemotherapy and those that did not (▨, $n = 5$). Box plot showing the 10th, 25th, 50th (median), 75th and 90th percentiles. $*P<0.02$; $\dagger P<0.02$.

**Table III** Relationship between P-glycoprotein and intracellular DNR or DOL [mean ± s.d. ($n$)]

| | *P-glycoprotein* | |
|---|---|---|
| | *Positive* | *Negative* |
| DNR (ng h $10^{-6}$ cells $mg^{-1}$ DNR) | 1.22 ± 0.77 (4)[a] | 2.17 ± 2.14 (8)[a] |
| DOL (ng h $10^{-6}$ cells $mg^{-1}$ DNR) | 0.26 ± 0.35 (4)[b] | 0.44 ± 0.44 (8)[b] |

[a] $P>0.3$. [b] $P>0.3$

present in the cells of patients with acute leukaemia. In view of our results it appears that the presence and the activity of this enzyme in leukaemic cells must be low. Incubating the leukaemic cell line CEM with DNR over 4 h did not produce any measurable DOL, confirming the lack of or extremely low level of daunorubicin reductase in these cells (unpublished results). Furthermore, when the metabolite DOL was incubated with CEM cells, only 14% of the metabolite was accumulated compared with the amount of DNR that would be accumulated. Therefore, it appears that the differences between plasma and cellular concentrations of DNR are due to the inability of DOL to cross the cell membrane and the lack of daunorubicin reductase in the cells.

There have been few reports on the correlation of plasma and cellular DNR pharmacokinetics and clinical response. In the present study, no correlation between patient response and plasma pharmacokinetics was observed. The average (± s.d.) plasma half-life and plasma clearance of DNR for all patients were 11 ± 11 h and 238 ± 100 l $h^{-1}$ respectively, which is similar to the values obtained by Speth *et al.* (1987) and Kokenberg *et al.* (1988). Kokenberg *et al.* (1988) found that there were no differences between plasma DNR or DOL AUCs compared with patient response. They also reported no relationship between any other plasma pharmacokinetic parameter and patient response. In this study, an inconsistency was noted: patients that received a 25 mg $m^{-2}$ dose of DNR achieved only approximately one-third of the plasma AUC of DNR (170 ± 53) compared with those patients that received 50 mg $m^{-2}$ (517 ± 296). One explanation might be that patients receiving 50 mg $m^{-2}$ DNR also received Ara-C and VP16 in combination, while those receiving 25 mg $m^{-2}$ received prednisolone and vincristine in combination. This suggests that either the combination of Ara-C and VP16 increases the plasma AUC of DNR or prednisolone and vincristine decrease the plasma AUC of DNR. Nearly all chemotherapeutic protocols involve the use of more than one agent, however there is no literature available on the pharmacokinetic interactions between DNR and any other agent used in chemotherapeutic regimens.

In this study there was a significant difference in both cellular DNR and DOL levels in those patients who underwent complete remission compared with those that did not respond. This is in contrast to the report of Kokenberg *et al.* (1988), who found that there was no correlation between any pharmacokinetic parameter and response to therapy. One possible explanation for the differences is that Kokenberg *et al.* (1988) compared intracellullar concentrations at a single time point, whereas the cellular concentration for a 24 h period ($AUC_{0-24h}$) was analysed in this study. A recent study by Marie *et al.* (1993) showed similar findings to this study *in vitro*. They found increased cellular DNR concentrations in patients achieving complete remission compared with those not responding to treatment. Concerning the other drugs used in induction therapy, they were given to both responders and non-responders, and thus affect both groups equally. In spite of the variables, i.e. different drug concentrations, different chemotherapy regimens and different types of leukaemia, a significant difference was observed in the cellular drug concentration between patients responding and those not responding to chemotherapy, implying that the correlation is independent of these factors. We were unable to recruit more patients to extend this study owing to the change in the clinical practice of the treatment of acute leukaemia, DNR having been replaced by newer anthracycline analogues (idarubicin) and anthracenes (mitoxantrone).

To our knowledge, this is the first report investigating the relationship between Pgp and intracellular levels of DNR in patients. The cellular concentrations of DNR and DOL tended to be lower in those patients who were Pgp positive than in those who were not (Table III), but statistically there was no difference. Overexpression of Pgp might not be the sole explanation for the lower cellular DNR in patient leukaemic cells. Further studies are required before this can be determined. One possible reason for the lower cellular DNR in patient leukaemic cells could be the presence of non-Pgp mechanisms of resistance, such as that associated with the HL 60/ADR cell line (Marsh et al., 1986). In this drug-resistant cell line there was a decrease in intracellular drug concentration, but no detectable Pgp. Recently, Krishnamachary and Center (1993) have demonstrated the presence of another membrane protein which may be responsible for the decreased cellular drug accumulation present in the HL 60/ADR cell line. This membrane protein has been associated with the overexpression of the *MRP* gene, and this gene may play a role in patients with acute leukaemia who do not respond to treatment.

Previous studies examining the relationship between Pgp and patient response have shown conflicting findings. Chan *et al.* (1991) observed a correlation between Pgp and patient response. Twenty-six out of 31 non-localised neuroblastoma patients who were Pgp negative had a complete response to treatment, as compared with 6 of the 13 who were Pgp positive. Campos *et al.* (1992) had similar findings with acute non-lymphoblastic leukaemia in which complete remission rates were significantly lower in Pgp-positive patients (23/71, 32%) than in Pgp-negative patients (64/79, 81%). Marie *et al.* (1991) and Pirker *et al.* (1991) also found a correlation between Pgp (*mdr1* gene expression) and patient response. Marie *et al.* (1991) observed a complete remission of 67% in patients with undetectable *mdr1* expression, compared with 29% in patients with increased expression. Pirker *et al.* (1991) found the complete remission rate to be 89% in *mdr1* RNA-negative patients and 53% in *mdr1*-positive patients. In contrast to the above findings, Holmes *et al.* (1989) established that the overexpression of the Pgp gene was not an important mechanism in previously untreated AML. In that study elevated levels of *mdr1* were seen in two out of eight patients with untreated AML, five out of eight with refractory AML and four out of five patients with secondary AML. Rothenberg *et al.* (1989) observed that eight out of nine patients with ALL at presentation had low levels of *mdr1* mRNA. In five patients at primary relapse, none had evidence of *mdr1* overexpression and 3 out of 15 patients with multiple relapses had elevated *mdr1* expression. They concluded that Pgp might play a role in some cases of drug resistance and that other mechanisms of resistance must exist. We have found no significant relationship between Pgp and patient response. Of the patients in this study, 17 out of 21 were previously untreated. Twelve of these patients were Pgp negative, with nine achieving complete remission (75%), and five were Pgp positive (2/5 achieving CR, 40%). Of the four patients that were previously treated, two were Pgp positive and two were Pgp negative. None of these patients responded to treatment. Our findings are similar to those of Rothenberg *et al.* (1989), who showed low levels of Pgp at induction but higher levels of Pgp in multiple relapse patients.

In conclusion, a correlation between the intracellular DNR and DOL concentrations and patient response was observed in this study. The relationship between Pgp and intracellular drug concentrations was also examined. Although there was no statistical correlation between Pgp and intracellular drug concentrations, there was a tendency for patients who were Pgp positive to have decreased intracellular concentrations of DNR and DOL. A higher proportion of previously treated patients were Pgp positive, but no correlation was found between Pgp and patient response, suggesting that mechanism(s) of drug resistance other than Pgp are important in clinical resistance to DNR.

This work was supported by a NH&MRC grant. We would also like to thank Janet McLachlan for performing the P-glycoprotein assay and the staff of Wards 12D and 12H at Royal North Shore Hospital for their assistance in collecting patient samples. P. Galettis was enrolled in the Department of Biochemistry and Physiology, University of Technology, Sydney, and supported by a NSW Health Department Scholarship.

### References

ALBERTS, D.S., BACHUR, N.R. & HOLTZMAN, J.L. (1971). The pharmacokinetics of daunomycin in man. *Clin. Pharmacol. Ther.*, **12**, 96-104.

BRADLEY, G., JURANKA, P.F. & LING, V. (1988). Mechanism of multidrug resistance. *Biochim. Biophys. Acta*, **948**, 87-128.

CAMPOS, L., GUYOTAT, D., ARCHIMBAUD, E., CALMARD-ORIOL, P., TSURUO, T., TRONCY, J., TREILLE, D. & FIERE, D. (1992). Clinical significance of multidrug resistance P-glycoprotein expression on acute nonlymphoblastic leukemia cells at diagnosis. *Blood*, **79**, 473-476.

CHAN, H.S.L., HADDAD, G., THRONER, P.S., DEBOER, G., LIN, Y.P., ONDRUSEK, N., YEGER, H. & LING, V. (1991). P-glycoprotein expression as a predictor of the outcome of therapy for neuroblastoma. *N. Engl. J. Med.*, **325**, 1608-1614.

FELSTED, R.L. & BACHUR, N.R. (1982). Human liver daunorubicin reductases. In *Enzymology of Carbonyl Metabolism Aldehyde Dehydrogenase and Aldo keto Reductase*. Weiner, H & Wermuth, B. (eds) pp. 291-305. Alan R. Liss: New York.

FOJO, A., AKIYAMA, S., GOTTESMAN, M.M. & PASTAN, I. (1985). Reduced drug accumulation in multiply drug-resistant human KB carcinoma cell lines. *Cancer Res.*, **45**, 3002-3007.

HOELZER, D., THIEL, E., BODENSTEIN, H., PLAUMANN, L., BUCHNER, T., URBANITZ, D., KOCH, P., HEIMPEL, H., ENGELHARDT, R., MULLER, U., WENDT, F.-C., SODOMANN, H., RUHL, H., HERRMANN, F., KABOTH, W., DIETZFELBINGER, H., PRALLE, H., LUNSCKEN, CH., HELLRIEGEL, K.-P., SPORS, S., NOWROUSIAN, R.M., FISCHER, J., FULLE, H., MITROU, P.S., PFREUNDSCHUH, M., GORG, CH., EMMERICH, B., QUEISSER, W., MEYER, P., LABEDZKI, L., ESSERS, U., KONIG, H., MAINZER, K., HERRMANN, R., MESSERER, D. & ZWINGERS, T. (1984). Intensified therapy in acute lymphoblastic and acute undifferentiated leukemia in adults. *Blood*, **64**, 38-47.

HOLMES, J., JACOBS, A., CARTER, G., JANOWSKA-WIECZOREK, A. & PADUA, R.A. (1989). Multidrug resistance in haemopoietic cell lines, myelodysplastic syndromes and acute myeloblastic leukaemia. *Br. J. Haematol.*, **72**, 40-44.

HUFFMAN, D.H. & BACHUR, N.R. (1972). Daunorubicin metabolism in acute myelocytic leukemia. *Blood*, **39**, 637-643.

KARTNER, N. & LING, V. (1989). Multidrug resistance in cancer. *Sci. Am.*, **260**, 44-51.

KARTNER, N., SHALES, M., RIORDAN, J.R. & LING, V. (1983). Daunorubicin-resistant Chinese hamster ovary cells expressing multidrug resistance and a cell-surface P-glycoprotein. *Cancer Res.*, **43**, 4413-4419.

KOKENBERG, E., SONNEVELD, P., SIZOO, W., HAGENBEEK, A. & LOWENBERG, B. (1988). Cellular pharmacokinetics of daunorubicin: relationships with the response to treatment in patients with acute myeloid leukemia. *J. Clin. Oncol.*, **6**, 802-812.

KRISHNAMACHARY, N. & CENTER, M.S. (1993). The MRP gene associated with a non-p-glycoprotein multidrug resistance encodes a 190-kDa membrane bound glycoprotein. *Cancer Res.*, **53**, 3658-3661.

MA, D.D., SCURR, R.D., DAVEY, R.A., MACKERTICH, S.M., HARMAN, D.H., DOWDEN, G., ISBISTER, J.P. & BELL, D.R. (1987). Detection of a multidrug resistant phenotype in acute nonlymphoblastic leukaemia. *Lancet*, **i**, 135-137.

MARIE, J.P., ZITTOUN, R. & SIKIC, B.I. (1991). Multidrug resistance (*mdr1*) gene expression in adult acute leukemias: correlations with treatment outcome and *in vitro* drug sensitivity. *Blood*, **78**, 586-592.

MARIE, J.P., FAUSSAT-SUBERVILLE, A.M., ZHOU, D. & ZITTOUN, R. (1993). Daunorubicin uptake by leukemic cells: correlations with treatment outcome and *mdr1* expression. *Leukemia*, **7**, 825-831.

MARSH, W., SICHERI, D. & CENTER, M.S. (1986). Isolation and characterization of adriamycin-resistant HL 60 cells which are not defective in the initial intracellular accumulation of drug. *Cancer Res.*, **46**, 4053–4057.

PASTAN, I. & GOTTESMAN, M. (1987). Multiple-drug resistance in human cancer. *N. Engl J. Med.*, **316**, 1388–1393.

PAUL, C., LILIEMARK, J., TIDEFELT, U., GAHRTON, G. & PETERSON, C. (1989). Pharmacokinetics of daunorubicin and doxorubicin in plasma and leukemic cells from patients with acute nonlymphoblastic leukemia. *Ther. Drug. Monit.*, **11**, 140–148.

PIRKER, R., WALLNER, J., GEISSLER, K., LINKESCH, W., HAAS, O.A., BETTELHEIM, P., HOPFNER, M., SCHERRER, R., VALENT, P., HAVELEC, L. & 2 others (1991). MDR1 gene expression and treatment outcome in acute myeloid leukemia (see comments). *J. Natl Cancer Inst.*, **83**, 708–712.

ROTHENBERG, M.L., MICKLEY, L.A., COLE, D.E., BALIS, F.M., TSURUO, T., POPLACK, D.G. & FOJO, A.T. (1989). Expression of the mdr-1/P-170 gene in patients with acute lymphoblastic leukemia. *Blood*, **74**, 1388–1395.

ROWLAND, M. & TOZER, T.N. (1989). *Clinical Pharmacokinetics: Concepts and Applications*. Lea & Febiger: Philadelphia.

SPETH, P.A., LINSSEN, P.C., BOEZEMAN, J.B., WESSELS, H.M. & HAANEN, C. (1987). Leukemic cell and plasma daunomycin concentrations after bolus injection and 72 h infusion. *Cancer Chemother. Pharmacol.*, **20**, 311–315.